# The role of oral and prenatal healthcare providers in the promotion of oral health for pregnant women

**DOI:** 10.1186/s12884-023-05654-x

**Published:** 2023-05-03

**Authors:** Dania E. Al Agili, Zeinab I. Khalaf

**Affiliations:** 1 Department of Dental Public Health, Faculty of Dentistry, King Abdulaziz University, P.O. Box 80200, Jeddah, 21589 Saudi Arabia; 2 Department of Academic & Training Affairs, Jeddah Health Directorate, Ministry of Health Jeddah, Jeddah, Saudi Arabia

**Keywords:** Oral health, Pregnancy, Primary healthcare, Health services, Best Practices, Saudi Arabia

## Abstract

**Background:**

Hormonal alterations and lowered immunity during pregnancy aggravated by poor oral hygiene increase the risk of pregnant women of developing oral diseases. We conducted this cross-sectional study to examine the role of oral and prenatal health providers in promoting dental care for pregnant women attending primary healthcare centers (PHCs) in Saudi Arabia.

**Methods:**

An online questionnaire was sent to a random sample of women who attended PHCs in Jeddah, during 2018–2019. From a total of 1350 women who responded to our questionnaire, 515 women reported having a dental visit before pregnancy. These women comprised our study sample. Bivariate analyses and multiple logistic regression models were conducted to examine associations between oral practices of dental and prenatal health providers (exposures) and women’s utilization of dental care during pregnancy (outcome). Covariates included age, education (< 12 years of education, 12 years of education, and > 12 years of education), family income (≤ 5,000, 5,001–7,000, 7,001–10,000, and > 10,000 Saudi Riyals), health insurance (yes/no), nationality (Saudi Arabian/non-Saudi Arabian), and presence of dental problems, such as toothache, dental caries, gingival inflammation, and the need for dental extractions.

**Results:**

Only 30.0% of women were informed by a dentist during their dental visit before pregnancy about the importance of visiting a dentist during pregnancy. About 37.0% of women were asked about oral health, 34.4% were informed about the importance of dental care during pregnancy, and 33.2% had their mouths inspected by prenatal health providers. Women informed by dentists about the importance of dental visits during pregnancy were twice as likely (Odds ratio [OR]: 2.42, 95% confidence interval [CI]: 1.63–3.60) to visit a dentist during pregnancy. Women who were referred to dentists, had their mouth inspected, or were advised to visit a dentist during pregnancy by prenatal providers were 4.29 (95% CI: 2.67–6.88), 3.79 (95% CI: 2.47–5.82), and 3.37 (95% CI: 2.16–5.27) times as likely to visit a dentist during pregnancy.

**Conclusion:**

The partaking of oral and prenatal healthcare providers in evidence-based oral health promotion practices, antenatal-dental collaboration, and closing the referral loop increase pregnant women’s access to and utilization of preventive and treatment dental services.

## Background

The oral health of pregnant women is essential to good health and wellbeing [1]. Hormonal alterations and lowered immunity during pregnancy aggravated by poor oral hygiene increase the risk of pregnant women of developing oral diseases [2–5]. Gingival inflammation is usually encountered from the second trimester onward, and pre-existing periodontitis are exacerbated during pregnancy [6, 7]. Furthermore, dietary changes and consumption of cariogenic foods, reduced salivary flow, morning sickness, and inadequate oral hygiene practices in pregnant women can lead to dental caries and dental erosions [7–9]. Worldwide, 50% of pregnant women experience dental pain and 40% suffer from periodontal disease [10, 11]. In Saudi Arabia, 70–90% of pregnant women have at least one dental problem [12, 13]. Periodontal disease was the most (30–60%) prevalent dental problem followed by dental caries and toothache [30–40%] [12, 14, 15]. Periodontal disease during pregnancy is associated with adverse birth outcomes, such as preterm birth and low-birth weight [16, 17]. Pregnant women with untreated dental infections have higher odds of experiencing sepsis, pre-eclampsia, and miscarriages [18]. Oral disease in pregnant women is also associated with the development of early childhood caries in their children [19]. These unfavorable health conditions for the mothers and their children, and the fact that mothers are responsible for inculcating healthy habits in their children at an early age, make the restoration of an expectant mother’s oral health to a disease-free status a primary goal of public health programs [20].

Globally, most women do not seek dental care during pregnancy [21]. In Saudi Arabia, the delivery of health services is supervised and financed by the Ministry of Health [MOH] and oral healthcare is integrated into prenatal care [22]. Nonetheless, more than half of the pregnant women in Saudi Arabia completely avoid dental care during pregnancy and most access dental care only when they have toothache or bleeding gums [10, 12–15, 23]. The most reported barriers to the utilization of dental care during pregnancy were low awareness of the importance of oral healthcare, negative perceptions regarding the safety of dental treatment, and lack of perceived need for dental care [10, 13, 15, 23, 24].

The World Health Organization underlines the importance of oral health integration with maternal and child health for the effective prevention and control of oral disease [25]. Physicians and dentists must work together to help women initiate and maintain oral health care during pregnancy and through the life span [26]. Currently, there is a dearth in the literature exploring how both prenatal care providers and dentists can encourage pregnant women to seek oral health care during pregnancy. Therefore, we undertook this study to determine the prevalence of various oral health promotion practices undertaken by oral and prenatal health providers, and to understand their impact on the likelihood of women visiting a dentist during pregnancy.

## Methods

We used a cross-sectional study design to examine the utilization of dental services by women during pregnancy. The study was approved by both King Abdulaziz University Faculty of Dentistry Research Ethics Committee [070-07-20] and the Institutional Review Board at the Ministry of Health [20-591E]. Based on these ethical clearances, we received a formal letter from the Directorate of Health Services in Jeddah to facilitate our research and data collection. A cover letter stating the study purpose, procedures, and contact information was sent to all subjects and the actual participation of women in the study indicated their consent. All methods were carried out in accordance with relevant guidelines and regulations.

There are five public general hospitals in Jeddah, Saudi Arabia. Each hospital is linked to a network of primary healthcare centers (PHCs). All 46 PHCs which were active at the time of study were included in our study. These PHCs provide free preventive, treatment, and health promotion services to Saudi Arabian nationals and selected eligible populations, such as non-Saudi Arabian spouses or children of Saudi Arabian nationals. The study population included all women who visited the antenatal care clinics in all PHCs and had an expected due date in 2019. The total population of eligible women was 3024. A random sample of 2026 postpartum women, which represented two thirds of the original population, was selected to ensure that the study sample represented the target population. We took the list of postpartum women from each clinic and followed a systematic selection process whereby we skipped every third woman on the list of each antenatal clinic until we have gone through all the women from the clinic. Then we moved to the next clinic and continued the same process. To be included in the study, the women must have had a live birth, had an active phone number, and spoke Arabic. The total number of women who fulfilled the study inclusion criteria was 1,792. When we called these women to invite them to participate in our study, 79 declined participation, 307 did not answer calls or messages, and 1406 responded to our survey. Of these, 56 were unfinished. Out of the 1350 complete responses, 515 women who visited a dentist in the six months that preceded their pregnancy were included in this study. We chose this group of women for our analysis to allow us to examine the role of oral and prenatal health providers’ practices on subsequent dental visits of women during pregnancy.

We used SurveyMonkey online tool (http://www.surveymonkey.com) to create a structured quantitative survey. The questionnaire was adapted from the Pregnancy Risk Assessment Monitoring System (PRAMS) [27] and further questions were added to fully address our study aims. It was created in English and then translated from English to Arabic and the translation fidelity was established through a back-translation method [28]. The questionnaire was pretested for content validity among four dentists. Face validity was tested twice among a similar population. The first pretest was conducted to ensure that the questions were clear, culturally appropriate, and linguistically accurate. The questionnaire underwent a thorough revision, and the final version was uploaded on the SurveyMonkey portal. The online survey was pretested among a similar population to ensure clarity and ease of navigation and to estimate the length of time it will take them to complete it.

The questionnaire data included questions on women’s dental visits during pregnancy, oral health status, oral health promotion by dental and prenatal health providers, and sociodemographic factors. Because all the questions in the survey were mandatory for the respondents to answer them, we did not have any missing data in the study. The survey was conducted between February 2021 and January 2022. The data were exported from the SurveyMonkey file to IBM Statistical Package for Social Sciences software (SPSS), Version 28.0.0.0. for data management and analysis.

The participants were asked about their use of dental health services before and during pregnancy. The outcome of study was having a dental visit during pregnancy. The oral health promotion practices (exposures) performed by dentists included (1) Talking about the importance of dental visits during pregnancy, (2) Talking about the importance of regular dental visits, (3) Demonstrating tooth brushing, (4) Explaining ways to keep teeth and gums healthy, and (5) Talking about the importance of oral health care. The practices (exposures) performed by prenatal health providers included (1) Looking in the mouth, (2) Advising patient to visit a dentist during pregnancy, (3) Referring the patient to dental care, (4) Asking about oral health, (5) Talking about the importance of dental care during pregnancy, (6) Explaining ways to care for teeth and gums, (7) Handing out printed oral health educational material.

Covariates included having any dental problems before or during pregnancy such as toothache, dental caries, gingival inflammation, and the need for dental extractions. Demographic characteristics such as age, level of educational attainment (< 12 years of education, 12 years of education, and > 12 years of education), nationality (Saudi Arabian/non-Saudi Arabian), family monthly income in Saudi Riyals (SR) (≤ 5,000, 5,001–7,000, 7,001–10,000, and > 10,000 (1$=3.75 SR)) and having a medical insurance (yes/no) were recorded. Health insurance includes dental coverage in Saudi Arabia.

Descriptive statistics in the form of means and standard deviations for continuous variables and frequencies and percentages for categorical variables were calculated for our study sample. The overall prevalence of each oral health practice and the prevalence of these practices for each demographic characteristic were calculated. We used Pearson’s chi-squared test for categorical variables (education, family income, nationality, health insurance, and any dental problems) and Independent-Samples T-test for continuous variables (age), to examine bivariate associations between having oral health practices performed by dentists and prenatal care providers and demographic characteristics, and between these practices and women having a dental visit during pregnancy. Because of presence of multicollinearity between oral health practices of dentists and those of prenatal health providers, we used separate binomial logistic regression models to quantify the adjusted associations. All multivariable models were adjusted for age, education, family income, health insurance, nationality, and any dental problems. All statistical tests were two-tailed with type-I error rate set at 5%.

## Results

Table 1 shows the characteristics of our study sample (n = 515). The mean age was 32.1 years (Standard deviation [SD] = 4.4) and the majority (54.0%) of women had more than 12 years of education. Most (39.3%) women had a monthly income of up to 5,000 SR and only 14% made more than 10,000 SR per month. A high percentage (88.5%) of women did not have health insurance and 89.9% were Saudi nationals. About 90.0% of women had some dental problems, and of these, 47.9% visited a dentist during their pregnancy.

**Table 1:** Characteristics of women in the study sample, N = 515

Characteristics	Statistic
Age, mean (standard deviation)	32.1 (5.5)
Education, n (%)	
< 12 years	76 (14.8)
12 years	161 (31.3)
> 12 years	278 (54.0)
Income, n (%)	
≤ 5,000 SR	202 (39.3)
5,001–7,000	127 (24.7)
7,001–10,000	114 (22.1)
> 10,000	72 (14.0)
Health insurance, n (%)	
No	456 (88.5)
Yes	59 (11.5)
Nationality, n (%)	
Non-Saudi	52 (10.1)
Saudi	463 (89.9)
Any dental problems, n (%)	
No	52 (10.1)
Yes	463 (89.9)
Dental visit during pregnancy, n (%)	
No	267 (51.8)
Yes	248 (48.2)

We found that 29.9%, 44.7%, and 61.9% of the women were informed by their dentists about the importance of dental visits during pregnancy, regular dental visits, and oral health care, respectively. There was an inverse dose-response relationship between women’s education and the prevalence of each of the important dental practices discussed by the dentists, with women with the lowest level of education (< 12 years) being the group with whom the dentists discussed important oral health care practices the most. Saudi women compared to non-Saudi women had a higher prevalence of having their dentists advise them about the importance of dental visits during pregnancy (31.3% vs. 17.3%) and regular dental visits (46.2% vs. 30.8%). Women without dental problems had higher prevalence of dentists explaining the importance of dental visits during pregnancy (44.2% vs. 28.3%), discussing the importance of regular dental visits (65.4% vs. 42.3%), and demonstrating tooth brushing techniques (69.2% vs. 45.1%) to them as compared to those with dental problems (Table 2).

**Table 2:** Prevalence of oral health promotion practices of dentists by characteristics of women, (n = 515)

Characteristic	Talked about importance of dental visits during pregnancy, n (%)		Talked about importance of regular dental visits, n (%)		Demonstrated toothbrushing, n (%)		Explained ways to keep teeth & gums healthy, n (%)		Talked about importance of oral health care, n (%)	
	154 (29.9%)		230 (44.7%)		245 (47.6%)		244 (47.4%)		319 (61.9%)	
	Col n (%)	PR	Col n (%)	PR	Col n (%)	PR	Col n (%)	PR	Col n (%)	PR
Age^a^	0.043		-0.222		0.086		0.148		0.456	
Education^b^										
< 12 years	30 (19.5)	39.5	39 (17.0)	51.3	46 (18.8)	60.5^*^	44 (18.0)	57.9	51 (16.0)	67.1
12 years	51 (33.1)	31.7	70 (30.4)	43.5	80 (32.7)	49.7	78 (32.0)	48.4	99 (31.0)	61.5
> 12 years	73 (47.4)	26.3	121 (52.6)	43.5	119 (48.6)	42.8	122 (50.0)	43.9	169 (53.0)	60.8
Family income^b^										
≤ 5,000 SR	61 (39.6)	30.2	90 (39.1)	44.6	97 (39.6)	48.0	99 (40.6)	49.0	117 (36.7)	57.9
5,001–7,000 SR	40 (26.0)	31.5	62 (27.0)	48.8	58 (23.7)	45.7	55 (22.5)	43.3	82 (25.7)	64.6
7,001–10,000 SR	28 (18.2)	24.6	46 (20.0)	40.4	54 (22.0)	47.4	58 (23.8)	50.9	76 (23.8)	66.7
> 10,000 SR	25 (16.2)	34.7	32 (13.9)	44.4	36 (14.7)	50.0	32 (13.1)	44.4	44 (13.8)	61.1
Nationality^b^										
Non-Saudi	9 (5.8)	17.3^*^	16 (7.0)	30.8^*^	24 (9.8)	46.2	19 (7.8)	36.5	30 (9.4)	57.7
Saudi	145 (94.2)	31.3	214 (93.0)	46.2	221 (90.2)	47.7	225 (92.2)	48.6	289 (90.6)	62.4
Health insurance^b^										
No	136 (88.3)	29.8	200 (87.0)	43.9	214 (87.3)	46.9	212 (86.9)	46.5	283 (88.7)	62.1
Yes	18 (11.7)	30.5	30 (13.0)	50.8	31 (12.7)	52.5	32 (13.1)	54.2	36 (11.3)	61.0
Any dental problems^b^										
No	23 (14.9)	44.2^*^	34 (14.8)	65.4^**^	36 (14.7)	69.2^***^	31 (12.7)	59.6	37 (11.6)	71.2
Yes	131 (85.1)	28.3	196 (85.2)	42.3	209 (85.3)	45.1	213(87.3)	46.0	181 (88.4)	60.9

Abbreviation: PR- prevalence

^a^ Independent sample t-test of significance; ^b^ Pearson’s χ2 test of significance

* p < 0.05; ** p < 0.01; *** p < 0.001

We noted that 36.8% of women reported that their health providers asked them about their oral health, 33.2% looked in their mouth, 29.9% advised them to visit a dentist during pregnancy and 15.8% gave them printed educational material about oral health care. The prevalence of talking about the importance of dental care during pregnancy, explaining ways to care for teeth and gums, and giving educational material about oral health care was highest in the lowest educational group and it decreased with increasing level of education. Prenatal care providers discussed the importance of dental care during pregnancy more with Saudi women as compared to their non-Saudi counterparts (32.4% vs. 17.3%: Table 3). The prevalence of all prenatal oral health practices did not differ by the presence or absence of dental problems in women (*p* > 0.05).

**Table 3:** Prevalence of prenatal health providers’ oral health practices by maternal characteristics, (N = 515)

Characteristics	Looked in mouth, n (%)		Referred to dental care, n (%)		Advised to visit dentist during pregnancy, n (%)		Asked about oral health, n (%)		Talked about importance of dental care during pregnancy, n (%)		Explained ways to care for teeth and gums, n (%)		Handed out oral health educational material, n (%)	
	139 (33.2)		133 (31.7)		125 (29.8)		154 (36.8)		144 (34.4)		104 (24.8)		66 (15.8)	
	Col, n (%)	PR	Col, n (%)	PR	Col, n (%)	PR	Col, n (%)	PR	Col, n (%)	PR	Col, n (%)	PR	Col, n (%)	PR
Age^a^	-0.988		-1.555		-0.648		1.231		0.26		-0.245		-1.583	
Education^b^														
< 12 years	27 (19.6)	35.5	20 (17.7)	26.3	20 (16.9)	26.3	33 (21.4)	44.6	32 (20.1)	42.1^*^	28 (25.2)	36.8^**^	19 (26.0)	25.0^*^
12 years	45 (32.6)	28.0	33 (29.2)	20.5	34 (28.8)	21.1	49 (31.8)	36.3	55 (34.6)	34.2	34 (30.6)	21.1	20 (27.4)	12.4
> 12 years	66 (47.8)	23.7	60 (53.1)	21.6	64 (54.2)	23.0	72 (46.8)	34.3	72 (45.3)	25.9	49 (44.1)	17.6	34 (46.6)	12.2
Family income^b^														
≤ 5,000 SR	55 (39.9)	27.2	50 (44.2)	24.8	54 (45.8)	26.7	61 (38.9)	30.2	67 (42.1)	33.2	49 (44.1)	24.3	35 (47.9)	17.3
5,001–7,000 SR	37 (26.8)	2915	24 (21.2)	18.9	26 (22.0)	20.5	41 (26.1)	32.3	36 (22.6)	28.3	22 (19.8)	17.3	18 (24.7)	14.2
7,001–10,000 SR	31 (22.5)	27.2	27 (23.9)	23.7	24 (20.3)	21.1	36 (22.9)	31.6	39 (24.5)	34.2	28 (25.2)	24.6	15 (20.5)	13.2
> 10,000 SR	15 (10.9)	20.8	12 (10.6)	16.7	14 (11.9)	19.4	19 (12.1)	26.4	17 (10.7)	23.6	12 (10.8)	16.7	5 (6.8)	6.9
Nationality^b^														
Non-Saudi	11 (8.0)	21.2	10 (8.8)	19.2	12 (10.2)	23.1	11 (7.0)	21.2	9 (5.7)	17.3^*^	6 (5.4)	11.5	6 (8.2)	11.5
Saudi	127 (92.0)	27.4	103 (91.2)	22.2	106 (89.8.2)	22.9	146 (93.0)	31.5	150 (94.3)	32.4	105 (94.6)	22.7	67 (91.8)	14.5
Health insurance^b^														
No	125 (90.6)	27.4	99 (87.6)	21.7	104 (88.1)	22.8	138 (87.9)	30.3	142 (89.3)	31.1	97 (87.4)	21.3	66 (90.4)	14.5
Yes	13 (9.4)	22.0	14 (12.4)	23.7	14 (11.9)	23.7	19 (12.1)	32.2	17 (10.7)	28.8	14 (12.6)	23.7	7 (9.6)	11.9
Any dental problems^b^														
No	14 (10.1)	26.9	9 (8.0)	17.3	11 (9.3)	21.2	18 (11.5)	34.6	16 (10.1)	30.8	12 (10.8)	23.1	10 (13.7)	19.2
Yes	124 (89.9)	26.8	104 (90.0)	22.5	107 (90.7)	23.1	139 (88.5)	30.0	143 (89.9)	30.9	99 (89.2)	21.4	63 (86.3)	13.6

Abbreviation: PR, prevalence

^a^ Independent sample t-test of significance. ^b^ Pearson’s χ2 test of significance

^*^: p < 0.05; ^**^: p < 0.001

Table 4 shows the associations between dental and prenatal care providers’ oral health practices and dental visit during pregnancy. Among oral health providers’ practices, talking to women about importance of dental visits during pregnancy had a higher prevalence of dental visits during pregnancy as compared to women who were not informed (63.3% vs. 41.6%, *p* < 0.001). Women who were informed by their dentists about the importance of regular dental visits (54.3% vs. 43.2%, *p* < 0.05), those who were shown how to properly brush their teeth (53.1% vs. 43.7%, *p* < 0.05), and those who were educated about ways to keep their teeth and gums healthy (52.9% vs. 43.9%, *p* < 0.05) had higher prevalence of having a dental visit during pregnancy in comparison to their counterparts who were foregone these practices. Regarding the oral health practices performed by prenatal health providers, all oral health practices were strongly (*p* < 0.001) associated with women’s access to dental care during pregnancy.

**Table 4:** Associations between different practices of oral health performed by oral and prenatal health providers and women having a dental visit during pregnancy, N = 515

Oral health provider’s practice	Dental visit during pregnancy		Prevalence	Prenatal health provider’s practice	Dental visit during pregnancy		Prevalence
	No	Yes			No	Yes	
	(n, %)	(n, %)			(n, %)	(n, %)	
Talked about importance of dental visits during pregnancy				Looked in mouth			
No	211 (79.0)	150 (60.5)	41.6^**^	No	227 (85.0)	150 (60.5)	39.8^**^
Yes	56 (21.0)	98 (39.5)	63.6	Yes	40 (15.0)	98 (39.5)	71.0
Talked about importance of regular dental visits				Referred to dental care			
No	162 (60.7)	123 (49.6)	43.2^*^	No	238 (89.1)	164 (66.1)	40.8^**^
Yes	105 (39.3%)	125 (50.4)	54.3	Yes	29 (10.9)	84 (33.9)	74.3
Demonstrated toothbrushing				Advised to visit dentist during pregnancy			
No	152 (56.9)	118 (47.6)	43.7^*^	No	232 (86.9)	165 (66.5)	41.6^**^
Yes	115 (43.1)	130 (52.4)	53.1	Yes	35 (13.1)	83 (33.5)	70.3
Explained ways to keep teeth and gums healthy				Asked about oral health			
No	152 (56.9)	119 (48.0)	43.9^*^	No	213 (79.80)	145 (58.5)	40.5^**^
Yes	115 (43.1)	129 (52.0)	52.9	Yes	54 (20.2)	103 (41.5)	65.6
Talked about importance of oral health care				Talked about importance of oral health care during pregnancy			
No	111 (41.6)	85 (34.3)	43.4	No	206 (77.2)	150 (60.5)	42.1^**^
Yes	156 (58.4)	163 (65.7)	51.1	Yes	61 (22.8)	98 (39.5)	61.6
				Explained ways to care for teeth and gums			
				No	229 (85.8)	175 (70.6)	43.3^**^
				Yes	38 (14.2)	73 (29.4)	65.8
				Handed out oral health educational material			
				No	245 (91.8)	197 (79.4)	44.6^**^
				Yes	22 (8.2)	51 (20.6)	69.9

Pearson’s χ2 test of significance was used to test for associations: *: p < 0.05; **: p < 0.001

Tables 5 and 6 show the unadjusted and adjusted odds ratios of women’s utilization of dental care during pregnancy by different oral health practices of oral and prenatal health providers. Women who were informed about the importance of dental visits during pregnancy had 2.42 times the adjusted odds (95% CI: 1.63–3.60, *p* < 0.001) of visiting a dentist during pregnancy as compared to those who were never informed. Women who were referred to dental care by their prenatal health providers were 4.29 times as likely (95% CI: 2.67–6.88, *p* < 0.001) to visit the dentist during pregnancy as compared to those who were not referred. Women whose prenatal health providers looked in their mouths were nearly 4 times as likely (OR: 3.79, 95% CI: 2.47–5.82, *p* < 0.001) to visit a dentist while pregnant compared to those whose mouths were not checked.

**Table 5:** Unadjusted and adjusted logistic regression models of women’s utilization of dental care during pregnancy by dentists’ practices, N = 515

Oral health provider’s practice	Unadjusted OR (95% CI)	p-Value	Adjusted OR (95% CI)	p-Value
Talked about importance of dental visits during pregnancy^a^	2.462 (1.668–3.634)	< 0.001	2.419 (1.625–3.601)	< 0.001
Talked about importance of regular dental visits^b^	1.57 (1.11–2.22)	0.012	1.55 (1.08–2.21)	0.017
Demonstrated toothbrushing^c^	1.46 (1.03–2.06)	0.034	1.44 (1.01–2.06)	0.045
Talked about ways to keep teeth and gums healthy^d^	1.43 (1.01–2.03)	0.043	1.40 (0.98–1.99)	0.064
Talked about importance of caring for teeth and gums^e^	1.36 (0.95–1.95)	0.089	1.37 (0.95–1.97)	0.09

All multivariate models were adjusted for age, education, family income, health insurance, nationality, and any dental problems.

^a^ Hosmer & Lemeshow Chi-square = 1.98, p = 1.0; Pseudo R^2^ = 0.06; ^b^ Hosmer & Lemeshow Chi-square = 4.51, p = 0.8; Pseudo R^2^ = 0.03; ^c^ Hosmer & Lemeshow Chi-square = 7.87, p = 0.4; Pseudo R^2^ = 0.02; ^d^ Hosmer & Lemeshow Chi-square = 2.79, p = 1.0; Pseudo R^2^ = 0.02; ^e^ Hosmer & Lemeshow Chi-square = 2.04, p = 1.0; Pseudo R^2^ = 0.02.

**Table 6:** Unadjusted and adjusted logistic regression models of women’s utilization of dental care during pregnancy by practices of prenatal health providers, N = 515

Prenatal health provider’s practice	**Unadjusted OR (95% CI)**	**p-Value**	**Adjusted OR (95% CI)** ^**a**^	**p-Value**
Looked in mouth^a^	3.06 (2.32–4.03)	< 0.001	3.79 (2.47–5.82)	< 0.001
Referred to dental care^b^	4.19 (3.11–5.64)	< 0.001	4.29 (2.67–6.88)	< 0.001
Advised to visit dentist during pregnancy^c^	3.24 (2.43–4.33)	< 0.001	3.37 (2.16–5.27)	< 0.001
Asked about oral health^d^	1.92 (1.50–2.47)	< 0.001	2.83 (1.90–4.22)	< 0.001
Talked about importance of oral health care during pregnancy^e^	1.902 (1.47–2.45)	< 0.001	2.19 (1.48–3.24)	< 0.001
Explained ways to care for teeth and gums^f^	2.44 (1.81–3.28)	< 0.001	2.49 (1.59–3.89)	< 0.001
Handed out oral health educational material^g^	2.81 (1.94–4.09)	< 0.001	2.92 (1.70–5.03)	< 0.001

All multivariate models were adjusted for age, education, family income, health insurance, nationality, and any dental problems

a Hosmer & Lemeshow Chi-square = 4.70, p = 0.8; Pseudo R2 = 0.11; b Hosmer & Lemeshow Chi-square = 8.10, p = 0.4; Pseudo R2 = 0.11; c Hosmer & Lemeshow Chi-square = 5.12, p = 0.7; Pseudo R2 = 0.09; d Hosmer & Lemeshow Chi-square = 11.82, p = 0.1; Pseudo R2 = 0.08; e Hosmer & Lemeshow Chi-square = 4.48, p = 0.8; Pseudo R2 = 0.05; f Hosmer & Lemeshow Chi-square = 11.52, p = 0.2; Pseudo R2 = 0.06; g Hosmer & Lemeshow Chi-square = 2.68, p = 1.0; Pseudo R2 = 0.05

## Discussion

To our knowledge, this is the first study in the region that examines and provides a strong understanding of the role of oral health practices, by both dental and prenatal health providers, in promoting dental care during pregnancy. This study added valuable insights to the existing scholarly literature, which found an increased utilization of dental visits during pregnancy when prenatal healthcare providers advise pregnant women to seek dental care. Our study analyzed specific oral health promotion practices by both prenatal healthcare providers and dentists and their respective impacts on the likelihood of women seeking dental care during pregnancy. Moreover, our study suggests that this likelihood of pregnant women seeking dental care will be higher when both prenatal healthcare providers and dentists encourage women to seek dental care during pregnancy.

We found that only half (48%) of the women who reported having any dental problems went to the dentist during their pregnancy. We also found that only 30% of the women who visited a dentist before pregnancy reported that their dentists talked to them about the importance of dental visits during pregnancy. This dental practice, however, was the most successful in persuading pregnant women to continue their dental care during pregnancy. Although multiple randomized controlled trials demonstrated that dental care during pregnancy is important and safe [29–31], and despite the publication of evidence-based guidelines advocating for the provision of oral health care during pregnancy [32, 33], dentists remain undecided about treating pregnant women. Insufficient knowledge in the care of pregnant women, uncertainties about the safety of dental treatment during pregnancy, fear of liability, and lack of inter-professional collaboration were some of the barriers to the provision of dental care to pregnant women reported by dentists [34–36]. An oral health pregnancy day initiative by the University of Detroit Mercy School of Dentistry which trained dental students in perinatal oral health guidelines and supervised their dental treatment of pregnant women, resulted in increased students’ knowledge, exposure, and comfort level in treating pregnant women [37]. Therefore, providing prenatal oral health training to dental students and increasing their exposure to pregnant patients facilitate access of pregnant women to oral health care.

Our findings also showed that women who were referred to dental care or had their mouth checked by a healthcare provider were four times more likely to visit a dentist during pregnancy. Moreover, advising expectant mothers to visit a dentist during pregnancy and asking them about their oral health increased their likelihood of visiting a dentist during pregnancy by three-folds. The Oral Health Care During Pregnancy Expert Workgroup (2012) recommended that prenatal health providers ask their pregnant patients oral health screening questions and check their mouths for problems during the first prenatal visit [38]. Our findings agreed with an analysis of the Maternal and Infant Health Assessment population-based survey of postpartum women in California, USA which found a two-fold increase in the prevalence of dental visits during pregnancy when the medical providers asked pregnant women about their oral health or suggested that they see a dentist. However, only 42% of the health providers talked to the women about oral health, while 26% suggested they see a dentist [39]. Although many prenatal health providers are aware of the need for dental care during pregnancy, most do not screen their pregnant patients for oral health nor refer them to a dentist [40]. This is unfortunate since they are usually the first point of contact when women get pregnant. Therefore, teaching medical students about the oral health care of pregnant women and exposing residents in family medicine and obstetrics and gynecology residency programs to prenatal oral health practice guidelines are important for improving access of pregnant women to early dental intervention and prevention.

Interestingly, we found that dentists are less likely to practice oral health promotion with women who had dental problems compared to those without dental problems. The need to complete the necessary dental treatment for those with dental problems and the limited time available for patients’ care in PHCs settings may explain this finding. Educating pregnant women about the importance of good oral heath during pregnancy, demonstrating proper tooth brushing and flossing, and providing professional prophylaxis are important in establishing a healthy oral environment in expectant mothers, and can be vital in improving perinatal outcomes and maternal and child dental health [41]. The role of dental hygienists in educating patients and preventing oral disease cannot be overemphasized.

Our study findings show that most of the examined oral health promotion practices are valuable in promoting dental visits during pregnancy, albeit to various extents. The dental practice of informing women of childbearing age that they should seek dental care during pregnancy is sufficient to encourage cautious pregnant women to obtain dental care during pregnancy. Likewise, prenatal health providers should routinely ask expectant mothers about their oral health, inspect their mouths, and refer them to dental care. Most women in Saudi Arabia trust their gynecologists and dentists equally when it comes to recommendations for dental treatment during pregnancy [13]. Therefore, interprofessional training in prenatal oral health guidelines should be designed and implemented to enable effective collaboration between dental and prenatal health providers and improve oral and prenatal health outcomes. Mandatory training policies should be put in place by the institution’s administration to ensure that all healthcare providers who are involved in the care of pregnant women are informed and integrating these evidence-based best practices into their routine clinical practices. This team-based approach can increase the number of pregnant women seeking dental care during pregnancy.

Currently, pregnant women at PHCs are referred to dental clinics for dental examination and education through a written referral form. Despite this, less than half of the study women accessed dental care during pregnancy. On the one hand, physicians may be non-compliant with the required dental referral. On the other hand, the limited number of dentists working in PHCs relative to the population served, shortages in hygienists and dental educators, and the lack of supportive services prohibit these centers from meeting the women’s demand for dental care [42]. Pregnant women referred to dental clinics must either wait a long time to be seen on the same day during a fully scheduled dental clinic or forgo dental care altogether if they are expected to schedule their own dental appointments. In addition, the limited availability of dental appointments via the “Sehhaty” digital health application and potential technical difficulties may constitute other factors that deter pregnant women from seeking dental care at PHCs. Hence, the employment of case coordinators is highly recommended for effective dental referrals and continuity of dental care [42–44]. Moreover, modifying the current electronic medical record system to support an electronic referral form will facilitate coordinated patient care and effective monitoring to close the referral loop [45].

Limitations of our study include the inherent characteristics of cross-sectional study designs. We are unable to establish that the increase in utilization of dental care by women during pregnancy is caused by prenatal health providers’ oral health promotion. Moreover, since the data were collected from pregnant women after delivery, there could be a potential for recall bias within the respondents, however, an event like a visit to a dentist during pregnancy is unlikely to be forgotten. Although under-coverage bias is a potential limitation, we do not feel that this limitation had a measurable influence on the validity of our study findings. Our study’s methodological rigor, its relatively large sample size and coverage of all PHCs in the city, and its high response (75%) and low exclusion rates (7%) make this study robust and our findings highly plausible. Finally, our study findings may not be generalizable to other pregnant women from more affluent sociodemographic backgrounds who attend private prenatal healthcare clinics in Jeddah. However, we do believe that our findings can be generalizable to all PHCs in the country and are extremely useful to the dental and obstetric community and to primary healthcare physicians worldwide.

The current reform in the Saudi healthcare delivery system and the new model of care through the Safe Birth System of Care (SOC) supports women from pre-marriage through pre-conception to post-delivery to promote safe pregnancies and healthy infants [46, 47]. Considering the enormous evidence linking poor oral health in expectant mothers to negative maternity and child health outcomes, it is compelling to include oral health as a basic component of SOC. This reform also underscores healthcare education and training. Therefore, training in the practice guidelines for the dental management of pregnant women [32, 33] should be compulsory for all health providers, new hires, or veterans, who are involved in the provision of care to pregnant women. Additionally, the support of key stakeholders in the healthcare system in Saudi Arabia will help women initiate and maintain oral health care during pregnancy and through their lifespan. Primarily, the Saudi Central Board for Accreditation of Health Care Institutions [48] recommends the administration of a well-structured preventive dental education program for pregnant women in PHCs as one of its standards for the Dental and Oral Health Chapter (Chap. 5.3) [49]. Furthermore, the Public Health Authority (Wegaya) should emphasize, as part of its National Preventive Guidelines for Periodic Health Examination, oral health examination and prevention of oral diseases for women of childbearing age and pregnant women. Finally, key performance indicators, such as antenatal dental referral, the formulation of a dental care plan for the referred pregnant woman, and dental prophylaxis should be established and monitored by institutional clinical authorities. These engagements ensure effective antenatal-dental collaboration and promote healthy pregnancy outcomes and a future generation of children free of oral disease.

## Conclusions

In summary, our findings serve as an initial step in the development of evidence-based interventions to foster antenatal-dental collaboration and well-organized integration of oral health care into prenatal care. Further research is needed to better understand the facilitators and barriers of integrated prenatal oral health care systems. Understanding and overcoming these barriers will inform and support Saudi Arabia’s health reforms in making significant progress towards achieving its healthcare goals of equitable access to affordable quality dental care by 2030.

## Data Availability

The datasets used and/or analyzed during the current study will be made available from the corresponding author on reasonable request.

## Abbreviations

PHC: Primary healthcare center
OR: Odds ratio
CI: Confidence interval
PRAMS: Pregnancy Risk Assessment Monitoring System
SR: Saudi Riyals
SD: Standard deviation
SOC: Safe Birth System of Care
